# An assessment of equity in the distribution of non-financial health care inputs across public primary health care facilities in Tanzania

**DOI:** 10.1186/s12939-017-0620-0

**Published:** 2017-07-11

**Authors:** August Kuwawenaruwa, Josephine Borghi, Michelle Remme, Gemini Mtei

**Affiliations:** 1 Ifakara Health Institute, Plot 463, Kiko Avenue Mikocheni, P.O. Box 78 373, Dar es Salaam, Tanzania; 2 Department of Global Health and Development, London School of Hygiene & Tropical Medicine, London, UK

**Keywords:** Equity, Distribution, Health care inputs, Tanzania

## Abstract

**Background:**

There is limited evidence on how health care inputs are distributed from the sub-national level down to health facilities and their potential influence on promoting health equity. To address this gap, this paper assesses equity in the distribution of health care inputs across public primary health facilities at the district level in Tanzania.

**Methods:**

This is a quantitative assessment of equity in the distribution of health care inputs (staff, drugs, medical supplies and equipment) from district to facility level. The study was carried out in three districts (Kinondoni, Singida Rural and Manyoni district) in Tanzania. These districts were selected because they were implementing primary care reforms. We administered 729 exit surveys with patients seeking out-patient care; and health facility surveys at 69 facilities in early 2014. A total of seventeen indices of input availability were constructed with the collected data. The distribution of inputs was considered in relation to (i) the wealth of patients accessing the facilities, which was taken as a proxy for the wealth of the population in the catchment area; and (ii) facility distance from the district headquarters. We assessed equity in the distribution of inputs through the use of equity ratios, concentration indices and curves.

**Results:**

We found a significant pro-rich distribution of clinical staff and nurses per 1000 population. Facilities with the poorest patients (most remote facilities) have fewer staff per 1000 population than those with the least poor patients (least remote facilities): 0.6 staff per 1000 among the poorest, compared to 0.9 among the least poor; 0.7 staff per 1000 among the most remote facilities compared to 0.9 among the least remote. The negative concentration index for support staff suggests a pro-poor distribution of this cadre but the 45 degree dominated the concentration curve. The distribution of vaccines, antibiotics, anti-diarrhoeal, anti-malarials and medical supplies was approximately proportional (non dominance), whereas the distribution of oxytocics, anti-retroviral therapy (ART) and anti-hypertensive drugs was pro-rich, with the 45 degree line dominating the concentration curve for ART.

**Conclusion:**

This study has shown there are inequities in the distribution of health care inputs across public primary care facilities. This highlights the need to ensure a better coordinated and equitable distribution of inputs through regular monitoring of the availability of health care inputs and strengthening of reporting systems.

**Electronic supplementary material:**

The online version of this article (doi:10.1186/s12939-017-0620-0) contains supplementary material, which is available to authorized users.

## Background

Inequalities in health outcomes remain widespread [3, 7, 13, 41]. In emerging market economies, for example, the rate of infant mortality is twice as high in poor households than better-off households [3]. To try and improve equity in outcomes, there is growing commitment to pursuing universal health coverage in low and middle-income countries, which includes a fundamental focus on equitable access to quality health care, without the risk of financial hardship [19, 48, 62]. To date, benefit incidence studies have generally found that the distribution of government health budgets tends to be pro-rich, with the better-off having better access to publicly-funded health services [55]. This is partly due to the concentration of health care inputs (funds, staff, medical supplies, drugs and equipment) at facilities located in urban areas that are more accessible to wealthier groups [9, 64]. Similarly, a study in Tanzania found that 20% of the population with the fewest health workers per capita had only 8% of health workers, compared to 46% in the 20% of the population with the most health workers [39]. In order to enable greater equity in access to health services, a more equitable distribution of health care inputs based on relative population health risks and need for health care is required [51, 55]. Indeed, a relative lack of inputs (including skilled staff, essential drugs and diagnostic equipment) in lower level facilities serving poorer populations is one of the factors generating a pro-rich distribution of health care service utilisation [26].

The existing literature on the distribution of public health care inputs in low and middle income countries has mainly considered equity in relation to resource allocation from the national to the district level, based on resource allocation guidelines [2, 4]. Countries typically base the allocation of health care funds to local government authorities on needs-based allocation formulae that include population size, demographic composition, levels of ill health and socio-economic status; although in some cases budgets are based on allocations in previous years (historical budgets) [2, 17, 31, 43, 58]. The allocation of other health care inputs such as human resources may also be based on need [14, 21], such as the amount and scope of services delivered at each facility informed by expert opinion; the ratio of health workers to population [14, 16, 21]; the level of facility use per year [16, 29].

While there has been some attention to the way inputs are allocated from the central to the local government level and the availability of guidelines to support such distributions, there is limited international evidence on equity in the distribution of health care inputs from the local government level down to health facilities. A study in Kenya examined the distribution of health care inputs at facility level in relation to socio-economic status [55], but such information is not available from other settings. Guidelines for how to allocate inputs across facilities within geographic areas are not always available, with potential for variation across local government authorities in the approach used [60]. Understanding how health care inputs get allocated from local government to primary health care facilities is important as it determines service availability at the population level, and will impact on service coverage among different socio-economic groups.

This paper aims to assess equity in the distribution of health care inputs across public primary health facilities at the district level in Tanzania. Specifically, we focus on the distribution of human resources, drugs, contraceptive commodities, medical equipment and medical supplies among public primary care facilities. We then discuss the implications of our findings for future resource allocation decisions in Tanzania, as well as other low and middle-income countries facing similar inequities.

## Methods

### Study setting

Decentralization has been one of the most important health sector reforms in Tanzania since the early 2000s, which led to the transfer of autonomy from the central government to local authorities (districts) of which there are currently 169 [18]. There are two main modes of financing districts that are used in this context: block grants and basket funding. Block grants are funds generated from general tax revenue, while basket funds come from external sources that are pooled at the central level [28, 43, 45]. A needs-based formula was developed by the Ministry of Health for the allocation of financial resources from the health basket fund and block grant to the district councils, based on the following criteria; age and sex-weighted population (50%); poverty levels (15%); an index of mileage to and within the local government area (15%); and burden of disease, to incorporate under-five and adult mortality rates plus any others available (20%) [28]. Recently, the government has adopted a performance-based approach implying that part of the basket distribution would be contingent on local government authorities (LGAs) meeting certain conditions [34]. Districts also receive revenue from cost sharing funds, which include user fee revenue from facilities as well as premium contributions for the community health fund (a form of community-based health insurance). Districts are also allocated a government matching grant equal to community health fund member contributions collected by each district [38, 54]. While this provides an incentive for districts to generate community health fund revenue, it cannot be described as an equitable distribution mechanism as relatively poor districts are less able to generate these contributions in the first place [38].

Much of the recruitment and distribution of health personnel is done at the central level, although regions and districts have the mandate to identify and inform the Ministry to deploy new staff to fill vacancies [36]. Staffing norms vary by level of care, for example, a public dispensary requires 15–20 staff, consisting of clinical and non-clinical support staff. Health centres are required to have between 39 and 52 staff [35].

The ordering of drugs and medical supplies is done quarterly by facilities using a system known as Integrated Logistic System (ILS) through the District Medical Office (DMO) to the Medical Stores Department (MSD), which in turn distributes facility-specific packs directly to the facilities [33, 52]. However, the medicine supply chain has been facing a number of challenges including insufficient availability of drugs at MSD to fulfil orders, long ordering cycles and late delivery of medicines and medical supplies [60]. Local government authorities can also use their own funds to procure medicines from alternative suppliers in case of medicine stock-outs at the MSD level. Districts have being allocating about 30–33% of revenue from the community health fund to public primary health care facilities for minor repair and the local purchase of drugs. Vaccines are allocated directly to facilities through the Expanded Programme for Immunisation.

While poverty is taken into consideration in the allocation of central government funds and basket funds from donors, the equity implications of existing staff allocation mechanisms and drug procurement are less clear. Hence this study seeks to examine how equitable the distribution of health care inputs is across facilities at district level.

### Study design

This is a quantitative assessment of equity in the distribution of health care inputs at the primary health care facility level within districts. By taking the health facility as the unit of analysis, rather than a geographic area, the focus is on resource distribution across service providers, thereby capturing equity of public and external resource allocation.

#### Study area and facilities

The study was carried out in three districts in Tanzania, an urban district within Dar es Salaam region (Kinondoni District), and two rural districts in Singida region (Singida Rural and Manyoni District). These districts were purposively selected to provide a contrast between urban and rural areas, and because they were implementing primary care reforms, such as introducing community health insurance and/or constructing and upgrading primary care facilities as part of the Primary Health Care Development Programme (Additional file 1: Annex 3). Additional file 1: Table S1 provides some relevant district information. The urban district (Kinondoni) has the highest population growth rate per year; almost double that of the other two districts. All districts have an average outpatient visit rate per capita per year of between 0.7 and 0.8.

A total of 69 public primary care facilities were sampled, (11 health centres and 58 dispensaries) representing 60% of facilities in Manyoni, 50% in Singida and 33% in Kinondoni. All newly constructed facilities (completed within the previous 12 months) were included. The remaining facilities were randomly selected, with a probability of selection proportional to the size of their catchment population.

### Data sources

#### Inputs

A facility survey was administered at each of the sampled facilities in the three districts between February and March 2014. This involved a series of questions to the facility in-charge relating to staffing levels by cadre, and facility management. The interviewers also did observations of current stocks for equipment, drugs and medical supplies and to review the availability of each on the day of the survey. The list of drugs, medical supplies and equipment were drawn from the World Health Organisation’s (WHO) service readiness measurement tools [45] and comprised 37 drugs, 10 medical supplies and 15 items of equipment (see Additional file 1: annex for a full list of items).

#### Equity

We measured the distribution of inputs in relation to: (i) the wealth level of patients using the facilities, which was taken as a proxy for the wealth of the population in the catchment area; and (ii) facility distance from the district headquarters.

For the first measure, data came from an exit survey that was administered to clients who received health care services at the sampled facilities. Clients were randomly sampled based on those who came for one of the following outpatient services: for antenatal care, child vaccination, self malaria, self cough, self diarrhoea, child malaria, child cough, or child diarrhoea, with a target of 10 per facility. A total of 729 patients were interviewed (242 in Kinondoni; 245 in Singida and 242 in Manyoni District) after using health services at the facility. This exit survey tool solicited information on household asset ownership and housing characteristics.

For the second measure, data on the distance between the facility and the district headquarters were derived from district transport officers’ record books.

### Measurement of inputs

We generated indices to measure the availability of staff, drugs, medical supplies and equipment at facilities. We created four indices related to facility staffing levels per 1000 population, one for each of three potential cadres: staff in the clinical cadre (including medical officers, assistant medical officers, clinical officers and clinical assistant), staff in the nursing cadre (including nurse officers, anaesthetists, registered nurses, nurse midwives, public health nurses and maternal and child health aides) and support staff (including medical attendants and health attendants). Support staffs are mainly holders of an ordinary secondary school certificate and have a pre-nursing certificate from a recognized institution. An index of total staff available per 1000 population was estimated by combining all items included in each of the three cadre-level indices. To measure staff per 1000 population, total staffs were divided by the facility catchment population and multiplied by a thousand. Catchment population data was extracted from the recent census which was conducted by the National Bureau of Statistics in Tanzania [42].

We grouped 37 drugs into 8 categories based on their therapeutic use in the clinical care guidelines of the Ministry of Health and Social Welfare, namely; anti-malarials (3); oxytocics (3); anti-hypertensives (4); antiretroviral therapy (ART) drugs (8); vaccines (6); antibiotics (6); anti-diarrhoeal (2) and others which includes normal saline, savlon, povidone iodine, eye drops and Rifampin, Isoniazid, Pyrazinamide and Ethambutol (RHZE) for tuberculosis treatment(5). We generated indices for each as well as an overall index by combining the above categories. Drugs were coded as 1 if available on the day of the survey and 0 if they were not available on the day of the survey. Indices were estimated as mean scores across all items within a category.

Equipment and medical supplies were coded as 1 if they were available or 0 if they were unavailable on the day of the survey. We generated an index of equipment availability as a mean score across all 15 equipment items, as well as an index of medical supply availability as a mean score across all 10 supply items, and an index of contraceptives containing 8 items.

Finally, we constructed an overall index for all non-staff inputs by generating a mean score across all non-staff items.

### Equity

We measured the wealth of patients attending the health facilities – considered a proxy of the catchment population – by means of a wealth index that was developed using principal component analysis [23, 57]. Ownership of a total of 28 items was measured based on their inclusion in previous studies [57] (Additional file 1: Annex 1). Facilities were ordered based on their index score, and grouped into five equally sized groups (quintiles) from poorest to least poor.

We measured distance between the facility and the district headquarters in kilometres. Facilities were ordered based on their distance and grouped into five equally sized groups from most remote to least remote [58].

### Data analysis

For each of the indices relating to the inputs, we estimated the mean index value for each of the quintiles of wealth/distance, as well as the standard deviation and tested for differences across wealth groups using the chi squared test. We estimated the pair-wise correlation coefficients between wealth and distance and health care inputs and assessed the statistical significance of the associations. Distance was inversely ranked implying that the closer facilities were better off compared to those which are far away.

In relation to wealth, we calculated the equity ratio, and concentration indices for each of the health care inputs (clinical staff, nursing staff, support staff, total staff; vaccination, antibiotics, anti-malarials, oxytocics, anti-hypertensive drugs, ART, anti-diarrhoeal; other drugs; equipment, medical supplies, contraceptives, and all non-staff inputs), to assess equity in their distribution. The equity ratio for a given index is calculated as the mean score in the least poor (least remote) quintile divided by the mean score in the poorest (most remote) quintile, with a ratio greater than one indicating pro-rich inequality. This index is chosen because of its simplicity, but has the disadvantage of not considering the full distribution across the population as a whole, and relying on each quintile’s equal size. Concentration indices and concentration curves are also generated as these are widely used in the literature on health inequalities and consider the distribution of inputs across the whole population [37, 59]. The concentration index is defined as twice the area between the concentration curve and the line of equality (the 45-degree line). The concentration curve plots the cumulative share of health care inputs by facilities ranked by the socio-economic status of their patients or by the distance from the headquarters. The distribution of a given health care input is considered pro-poor (pro-rich) if the concentration curve of a given health care input lies above (below) the line of equality, and if the concentration index is negative (positive). Dominance tests were used to explore the statistical significance of the differences between the concentration curves of health care inputs and the 45 degree line of equality. Dominance tests were conducted at 5% significance level using 19 quintile points and applying the multiple comparison approach (mca), as proposed in [44]. If the concentration curve for health care inputs used lies below the 45 degree line of equity, the 45 degree line of equity is said to dominate the concentration curve (D-).

### Consent and ethical approval

Ethical clearance for this study was obtained from the Ifakara Health Institute Institutional Review Board (IRB), the Tanzanian National Institute for Medical Research (NIMR) and the London School of Hygiene & Tropical Medicine (LSHTM) Research Ethics Committee. District managers and health facility in-charges were informed about the study and written informed consent was obtained from each participant.

## Results

### Staffing levels by cadre

Additional file 1: Table S2 presents a correlation matrix showing the association between the availability of health care inputs, the wealth index and distance variable. There was a borderline significant positive association between the quantity of nurses per thousand population and the wealth of the facility catchment population (the correlation coefficient was 0.35) (Additional file 1: Table S2). The association between the quantity of staff and distance showed a negative association except for supportive staff, but these were not statistically significant.

**Fig. 1:**
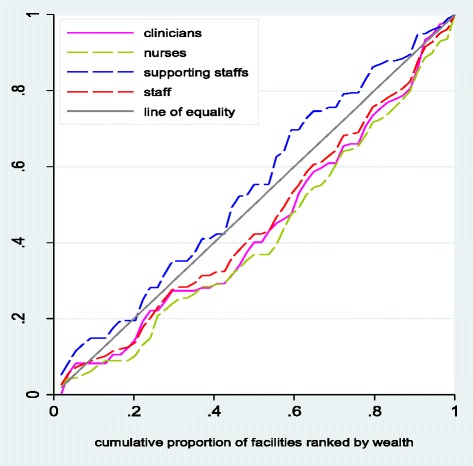
Concentration curves for total staff per thousand population in public primary facilities. Distribution of the staff per thousand population in public primary facilities using cumulative share of wealth/distance in Ikungi, Kinondoni, Manyoni and Singida district councils: Tanzania

Additional file 1: Table S3a and S3b present the distribution of health care inputs by wealth and distance quintiles. For both measures the distribution of staff is skewed towards the more advantaged (wealthier catchment areas and closer to the district headquarters). Facilities with the poorest patients (most remote facilities) have fewer staff per 1000 population than those with the least poor patients (least remote facilities) i.e. 0.6 staff per 1000 among the poorest, compared to 0.9 among the least poor; 0.7 staff per 1000 among the most remote facilities compared to 0.9 among the least remote. The distribution of clinical and nursing staff was pro-rich as demonstrated by the equity ratios and concentration indices (Additional file 1: Table S3a) and concentration curves (Fig. 1), with the distribution of nursing staff being the most pro-rich (CI: 0.122, p ~ 0.105 and CI: 0.167 p ~ 0.007). The negative concentration index (CI: −0.082, p ~ 0.331) for support staff suggests a pro-poor distribution of this cadre but the 45 degree dominated the concentration curve.

### Drugs

Vaccinations were the most widely available with almost all facilities reporting availability of vaccines on the day of the survey, irrespective of patient wealth and distance from the district headquarters, followed by anti-malarials at over 90% (Additional file 1: Tables S3a and S3b). While around half of facilities had essential antibiotics, anti- diarrhoeal and other drugs, facilities with poorer catchment populations were much less likely to stock oxytocics, anti-hypertensives or ARTs on the day of the survey than those with wealthier populations.

**Fig. 2:**
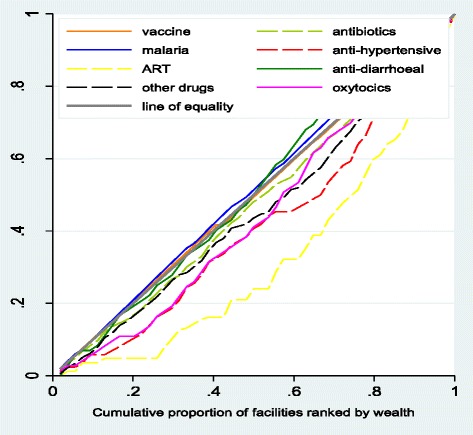
Distribution of drugs to public primary facilities Distribution of drugs to public primary facilities using cumulative share of wealth/distance in Ikungi, Kinondoni, Manyoni and Singida district councils: Tanzania.

There was a significant positive (negative) correlation between wealth (distance) and the availability of ARTs (p ~ 0.000), and a significant positive association between wealth and the availability of anti-hypertensives and other drugs (p ~ 0.000), but the association was not significant for distance (Additional file 1: Table S2). There was a non-significant negative (positive) correlation between wealth (distance) for anti-malarial drugs, and a significant negative association between vaccines and distance (p ~ 0.042). The distribution of vaccines, antibiotics, anti-diarrhoeal and anti-malarials was approximately proportional (non dominance), whereas the distribution of oxytocics (CI: 0.114, p ~ 0.008), ART’s (CI: 0.366, p ~ 0.000) and anti-hypertensive (CI: 0.179, p ~ 0.001) drugs was pro-rich (see Fig. 2 and Additional file 1: Table S3a). However, only the distribution of ARTs achieved dominance.

### Medical equipment, medical supplies and contraceptives

Availability of essential equipment also varied across facilities, from being present in just over 50% of the poorest facilities, to just under 80% of the least poor, with medical supply availability varying between similar ranges. Contraceptives were available in around half of all facilities sampled.

**Fig. 3:**
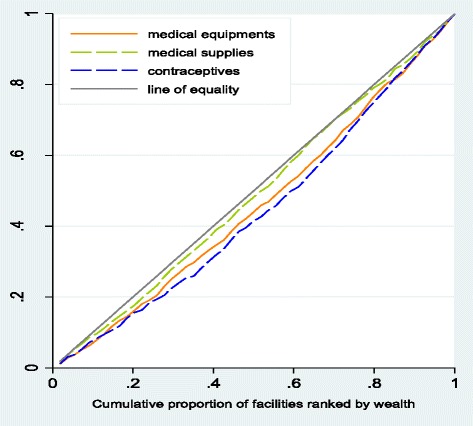
Distribution of medical equipment and medical supplies to public primary facilities. Distribution of medical equipment and medical supplies to public primary facilities using cumulative share of wealth/distance in Ikungi, Kinondoni, Manyoni and Singida district councils: Tanzania

There was a positive (negative) association between wealth (distance) and the availability of contraceptives, equipment and medical supplies, with contraceptives having the greatest association with wealth, and the availability of equipment having the greatest association with distance (Additional file 1: Table S2). Additional file 1: Tables S3a and S3b indicate that the concentration of medical equipment, supplies and contraceptives is higher in facilities serving least poor patients (least remote) than those servicing poorer patients (most remote). The distribution of contraceptives (CI: 0.117, p ~ 0.000) and medical equipment (CI: 0.087, p ~ 0.000) were pro-rich; however, there was non-dominance in both cases (Additional file 1: Table S3a and Fig. 3). The distribution of medical supplies was almost proportional (Additional file 1: Table S3a and Fig. 3).

### Overall inputs

**Fig. 4:**
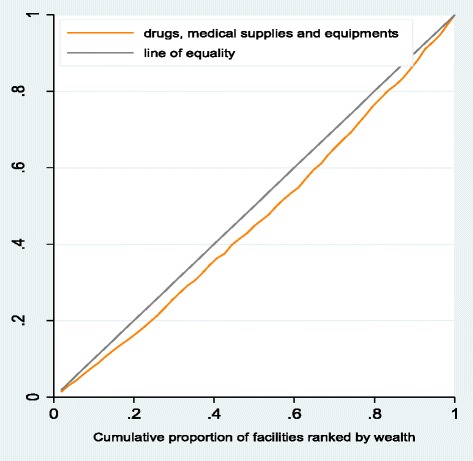
Overall distribution of drugs, medical supplies, equipment to public primary facilities. Distribution of drugs, medical supplies, equipment to public primary facilities using cumulative share of wealth/distance in Ikungi, Kinondoni, Manyoni and Singida district councils: Tanzania

Figure 4 depicts the overall distribution of inputs (excluding staff) in public primary health care facilities. The overall distribution of inputs excluding staff was marginally pro-rich (CI: 0.076, p ~ 0.000).

## Discussion

This study assesses the level of equity in the distribution of health care inputs at public primary health care facilities in Tanzania. We find a pro-rich distribution of nurses and clinical staff and of ARTs. The distribution of oxytocics, anti-hypertensive drugs and contraceptives were pro-rich although there was non-dominance. The distribution of vaccines, anti-malarials, anti-biotics and medical supplies was almost proportional.

This contributes to the limited evidence base on the equitable distribution of human resources at the facility level [64], and it is the second study, to our knowledge, to look at equity in the distribution of drugs, medical equipment and supplies from the district level down to the facility level [55].

Our findings reveal the greatest inequity in the distribution of staff in public primary health care facilities, with a concentration of staff in facilities serving least-poor populations, particularly the clinical and nursing staff needed to deliver services. Facilities serving the poorest patients appeared somewhat more likely to be served by support staffs, including medical attendants and health attendants, than those serving the least poor patients, though this was not supported by the dominance test. We also find a negative but not significant correlation between clinical and nursing staffing levels per 1000 population and distance from the district headquarters, a finding that is similar to previous studies that reported a concentration of health workers in urban areas in countries like Mali, Sudan, Uganda, Botswana, South Africa and Tanzania [55, 64]. Munga and Maestad [39] also found significant inequalities in the distribution of health workers per capita and inequities in the skill mix of health care staff in the districts.

The national norm stipulates that the lowest level primary health care facility (the dispensary) should be run by a clinical assistant (a secondary school graduate with 2 years of basic medical training), aided by an enrolled nurse (secondary school graduate with 2 years training in nursing care of minor ailments) [24, 35]. However, because of acute staff shortages, it not unusual to find a dispensary with neither a clinical assistant or a nurse; and instead being run by a health worker without any medical training [24, 53]. In our sample, 4% of facilities had no clinical or nursing staff. Individual career plans, existing salary levels, recruitment procedures and retention measures have led to an unequal distribution of the health workforce in Tanzania [24], as in many other countries in the region [50, 65]. While districts have some capacity to budget for staff, the distribution of resources is mainly centrally determined. Allowing providers to have greater autonomy to recruit locally, and pay for staff, may serve to address the current inequity, as has been successfully employed in other countries, such as Thailand [63, 65], along with incentives to work in rural areas. Initiatives have been undertaken by the government and other stakeholders to invest in the training and deployment of health care providers in order to reach people living in remote/marginalized areas; however there is limited evidence of their effectiveness [15, 47].

The availability of drugs, supplies, and equipment was generally higher than that reported elsewhere [55], across all socio-economic groups, with the overall distribution being marginally pro-rich, similar to Kenya, although the Kenyan study did not examine the distribution across drug types. In Tanzania, the greatest inequity was found in the distribution of ARTs, followed by anti-hypertensive drugs. These drugs can be expensive which may limit their affordability for facilities with less resources, hypertensive patients are less likely to seek care, which may be partly a result of limited drug availability, or indeed drug availability may be lower because of lower levels of demand [20, 30, 40]. The almost proportional distribution of anti-malarial drugs and vaccines suggests that government and donor initiatives to alleviate malaria through subsidised drugs and to ensure children have access to vaccination through an expanded immunisation programme may have played an important role [1, 25, 27, 56]. In 2006,Tanzania received USD 75 million from the Global Fund to Fight AIDS, Tuberculosis and Malaria to purchase artemisinin-based combination therapies (ACTs) for use in the public sector [32] and in 2011 weekly monitoring of facility level stock outs of anti-malarials by districts through mobile messaging was rolled out, SMS for Life [6]. For anti-malarials, however, there is some evidence suggesting that overall drug availability in the health system is pro-rich given the widespread availability of these drugs in the private retail sector that caters more to urban populations with higher purchasing power [10]. The distribution of medical equipment and contraceptives was also pro-rich, although there was non-dominance, but the distribution of medical supplies was almost proportional within the surveyed primary health care facilities.

In some cases, the inefficient ordering system might have affected the availability of certain drugs, medical equipment and supplies, and disproportionately disadvantage more remote facilities and those serving poorer populations [22].. Indeed, given the lower level staff cadres available at more disadvantaged facilities, their capacity to project the amount of inputs required for subsequent quarters is questionable and may further exacerbate the availability of essential medicines and supplies [24, 33]. Moreover, disease cases will vary by locality as well as season of the year, but not all staff have enough knowledge to predict such variation, and they will often rely on previous ordering records [8, 46].

A national incentive programme (results-based financing) is currently being scaled up and has the potential to affect the distribution of resources across facilities. Three quarters of the incentives earned by providers will be for facility use and can be reinvested in the procurement of essential drugs and supplies to address stock outs.

There is a need for health care inputs to be equitably allocated in order to ensure an optimal mix of complementary inputs at the point of service delivery, if countries are to achieve universal health coverage [11]. Tanzania serves as an interesting case, given the commitment to decentralisation of health care financing to the district level since the 1990s [18]. As in other countries that have adopted a decentralization policy, inconsistencies persist in terms of who governs and allocates each health care input (human resources, physical infrastructure, drugs, medical supplies and equipment) [12], which can lead to a sub-optimal mix of inputs that constrain the production of quality health services. As in several other countries, the type of medical equipment, supplies and drugs allocated to a facility is partly determined by the staff available to effectively use and prescribe these non-human resource inputs. Therefore, an inequitable distribution of staff between primary health care facilities will have further repercussions on the distribution of other health care inputs, and therefore on the health service outputs produced [61].

Data on community health needs and real-time health facility information also have a central part to play in enabling practitioners, managers and policy-makers to identify those in greatest need and to ensure that health care resources are used to maximize health improvement [49]. It is vital for planners to carefully review the process of allocating health care inputs in relation to community health needs. Given that populations are dynamic and that disease patterns change, the distribution of health care inputs should reflect such dynamics and the minimum package of services required per level of health care should respond to new emerging health care problems, including non-communicable diseases [5]. District health information management systems can be a vital tool in enabling planners to regularly review patients’ access, health care use and health care inputs available at facilities in order to enable facilities to offer appropriate needs-based services appropriate, and hence reducing inequities in access and use.

This study has a number of limitations. We only examined drug availability at a single point in time, rather than supplies over time. This could be an issue if facilities receive supplies at different time points. The assessment of the availability over time may have yielded different findings. Our estimates reflect the context of public primary facilities sampled from only three districts in the country and may not represent the situation nationally, nor does it reflect the distribution of resources across higher level facilities or those outside the public sector. However, our sample corresponds to a sizable share of all primary care public facilities in the districts sampled. While our sample comprises different levels of care (health centre and dispensary) the drugs, medical supplies and equipment examined would be expected to be available in all facilities. Our estimates of socio-economic status reflect the sample of patients interviewed at facilities, and may not represent the entire population in the facility catchment area, although this group is likely to be better off on average than the general population, this would be the case across all facilities. Further, our measure of wealth is a relative measure for the sampled population. In addition, not all essential drugs and supplies were captured, since the focus of the study was on maternal and child health services. That being said, this does reflect the majority of the case load at lower level facilities in most low and middle income countries. Unlike previous studies [55] we did not adjust our measures of concentration, to adjust for other facility level factors such as facility type, district, and remoteness.

## Conclusions

Access to and utilisation of affordable and quality primary health care services is essential for the move towards universal health coverage. The findings of this study have shown inequities in the distribution of health care inputs across primary care facilities and the need to ensure a better coordinated and pro-poor distribution to meet the needs of the populations served by these facilities. This includes the identification of shortages, regular monitoring of the availability of health care inputs and strengthening of reporting systems. Moreover, there is need for further research to model the health care resource inputs required at the sub-national level to meet existing population needs.

## Additional file

Additional file 1: Table S1. District Information. Basic information for Ikungi, Kinondoni, Manyoni and Singida district councils: Tanzania. **Table S2.** Correlation Matrix. Correlation Matrix of health care inputs and wealth/distance of health care facilities from district headquarter in kilometres. **Table S3a.** Descriptive Statistics by Wealth Quintiles. Descriptive Statistics of health care inputs by Wealth Quintiles. **Table S3b.** Descriptive Statistics by Distance. Descriptive Statistics of health care inputs by distance of health care facilities from district headquarter in kilometres. **Annex 1.** Household Ownership of Properties. Information on household ownership of properties which was used to develop wealth index using principal component analysis. **Annex 2.** Health Facility Survey tool. Health facility survey tool which was used to capture information on health care inputs (availability of staff, drugs, medical supplies and equipment at facilities) **Annex 3.** Health care reforms. Health care reforms which were being monitored and evaluated under Universal Coverage in Tanzania and South Africa (UNITAS) project. (DOC 312 kb)

## Abbreviations

ACTs: Artemisinin combination therapies
ADDOs: Accredited Drug Dispensing Outlets
ART: Antiretroviral therapy
CCHP: Comprehensive council health plans
CI: Concentration Index
DHIMS: District health information management system
DMO: District Medical Officer
ILS: Integrated Logistic System
LGAs: Local Governmental Authorities
MCH: Maternal and child health care
MFEA: Ministry of Finance and Economic Affairs
MoHSW: Ministry of Health and Social Welfare
MRDT: Malaria rapid diagnostic tests
MSD: Medical Store Department
MUAC: Middle-Upper Arm Circumference
NHIF: National Health Insurance Fund
PCA: Principal Component Analysis
SES: Socio-economic status
TZS: Tanzanian Shilling
UHC: Universal Health Coverage
USAID: United States Agency for International Development
WHO: World Health Organisation’s
