# Supplementary material for: An assessment of equity in the distribution of non-financial health care inputs across public primary health care facilities in Tanzania
Source: Int J Equity Health. 2017 Jul 11;16:124. doi: 10.1186/s12939-017-0620-0 (PMC5505032; doi:10.1186/s12939-017-0620-0)
Supplement: Additional file 1: Table S1. — District Information. Basic information for Ikungi, Kinondoni, Manyoni and Singida district councils: Tanzania. Table S2. Correlation Matrix. Correlation Matrix of health care inputs and wealth/distance of health care facilities from district headquarter in kilometres. Table S3a. Descriptive Statistics by Wealth Quintiles. Descriptive Statistics of health care inputs by Wealth Quintiles. Table S3b. Descriptive Statistics by Distance. Descriptive Statistics of health care inputs by distance of health care facilities from district headquarter in kilometres. Annex 1. Household Ownership of Properties. Information on household ownership of properties which was used to develop wealth index using principal component analysis. Annex 2. Health Facility Survey tool. Health facility survey tool which was used to capture information on health care inputs (availability of staff, drugs, medical supplies and equipment at facilities) Annex 3. Health care reforms. Health care reforms which were being monitored and evaluated under Universal Coverage in Tanzania and South Africa (UNITAS) project. (DOC 312 kb) [file 12939_2017_620_MOESM1_ESM.doc]

Table S1: District Information

|  | Kinondoni | Singida | Manyoni |
| --- | --- | --- | --- |
| Population^1^ | 1,775,049 | 476,121 | 296,763 |
| Population Growth rate per year | 4.3% | 2.4% | 2.3% |
| Area coverage in square kilometers^1^ | 531 | 12,164 | 28,620 |
| Total Public Health Centres | 3 | 5 | 4 |
| Total Public Dispensaries | 48 | 46 | 40 |
| Primary care facilities per 10,000 population | 0.29 | 1.07 | 1.48 |
| Outpatient visits (OPD) per capita | 0.8 | 0.7 | 0.7 |
| Primary health facilities surveyed |  |  |  |
| Health Centres | 3 | 4 | 4 |
| Dispensaries | 18 | 21 | 19 |

1 NBS, population and housing census 2013

Table S2: Correlation Matrix

| Variables | Wealth Index | Distance |
| --- | --- | --- |
| PCA | 1.000 |  |
| Distance | -0.536** | 1.000 |
| Facility Catchment Population | 0.627* | -0.408* |
| Clinical staff per 1000 population | 0.212 | -0.148 |
| Nurses per 1000 population | 0.350* | -0.187 |
| Support staff per 1000 population | -0.172 | 0.117 |
| Total staff per 1000 population | 0.217 | -0.119 |
| Vaccination availability index | 0.063 | -0.278* |
| Antibiotics availability index | 0.234 | -0.074 |
| Anti-malarial availability index | -0.197 | 0.071 |
| Oxytocics -availability index | 0.261 | -0.182 |
| Anti-hypertensive drugs availability index | 0.486* | -0.205 |
| ART availability index | 0.663* | -0.369* |
| Anti-diarrhoeal | -0.150 | 0.037 |
| Other drugs | 0.484* | -0.257 |
| Total drugs | 0.480* | -0.297 |
| Equipment availability index | 0.539* | -0.332* |
| Medical supplies available index | 0.131 | -0.147 |
| Contraceptive available index | 0.628* | -0.215 |
| Overall– excluding staff | 0.611* | -0.337* |

Pair-wise correlations test - * Significant at 10%; ** significant at 5%; *** significant at 1%

Table S3a: Descriptive Statistics by Wealth Quintiles

| Social economic status | Poorest | | Second Poorest | | Middle | | Second Richest | | Least poor | | Total | | Equity ratio | Concentration Indices | Dominance |
| --- | --- | --- | --- | --- | --- | --- | --- | --- | --- | --- | --- | --- | --- | --- | --- |
| Variables | n | Mean[std] | n | Mean[std] | n | Mean[std] | n | Mean[std] | n | Mean[std] | n | Mean[std] |  |  |  |
| Clinical staff per 1,000 population | 11 | 0.19[0.26] | 11 | 0.19[0.23] | 11 | 0.31[0.23] | 11 | 0.30[0.24] | 10 | 0.36[0.32] | 54 | 0.27[0.26] | 1.85 | 0.122* | D- |
| Nurses per 1,000 population, ** | 11 | 0.23[0.21] | 11 | 0.41[0.40] | 11 | 0.44[0.35] | 11 | 0.51[0.31] | 10 | 0.67[0.43] | 54 | 0.45[0.36] | 2.94 | 0.167** | D- |
| Support staff per 1,000 population | 11 | 0.22[0.21] | 11 | 0.26[0.24] | 11 | 0.31[0.37] | 11 | 0.20[0.19] | 10 | 0.16[0.19] | 54 | 0.23[0.25] | 0.73 | -0.082 | D- |
| Total staff per 1,000 population, * | 11 | 0.64[0.45] | 11 | 0.87[0.64] | 11 | 1.06[0.67] | 11 | 1.01[0.67] | 10 | 1.19[0.75] | 54 | 0.95[0.64] | 1.85 | 0.094* | D- |
| Vaccination availability index | 11 | 1.00[0.00] | 11 | 1.00[0.00] | 11 | 0.91[0.30] | 11 | 0.97[0.07] | 10 | 1.00[0.00] | 54 | 0.98[0.14] | 1.00 | 0.000 | Non-dominance |
| Antibiotics availability index , * | 11 | 0.53[0.15] | 11 | 0.67[0.20] | 11 | 0.59[0.17] | 11 | 0.73[0.15] | 10 | 0.67[0.22] | 54 | 0.64[0.19] | 1.26 | 0.044* | Non-dominance |
| Anti-malarial availability index, *** | 11 | 0.97[0.10] | 11 | 0.97[0.10] | 11 | 0.91[0.16] | 11 | 0.91[0.16] | 10 | 0.87[0.32] | 54 | 0.93[0.18] | 0.90 | -0.021 | Non-dominance |
| Oxytocics -availability index, *** | 11 | 0.24[0.16] | 11 | 0.49[0.17] | 11 | 0.46[0.23] | 11 | 0.55[0.27] | 10 | 0.53[0.32] | 54 | 0.45[0.25] | 2.21 | 0.114** | Non-dominance |
| Anti-hypertensive drugs availability index, *** | 11 | 0.21[0.15] | 11 | 0.43[0.20] | 11 | 0.27[0.18] | 11 | 0.50[0.30] | 10 | 0.60[0.34] | 54 | 0.40[0.27] | 2.86 | 0.179** | Non-dominance |
| ART availability index, *** | 11 | 0.09[0.17] | 11 | 0.22[0.23] | 11 | 0.30[0.43] | 11 | 0.55[0.28] | 10 | 0.81[0.27] | 54 | 0.39[0.38] | 9.00 | 0.366*** | D- |
| Anti-diarrhoeal index | 11 | 0.64[0.39] | 11 | 0.68[0.25] | 11 | 0.82[0.25] | 11 | 0.64[0.32] | 10 | 0.55[0.37] | 54 | 0.67[0.32] | 0.86 | -0.019 | Non-dominance |
| Other drugs, *** | 11 | 0.55[0.22] | 11 | 0.66[0.20] | 11 | 0.49[0.31] | 11 | 0.80[0.16] | 10 | 0.84[0.21] | 54 | 0.66[0.26] | 1.53 | 0.096** | Non-dominance |
| Total drugs , *** | 11 | 0.53[0.08] | 11 | 0.64[0.07] | 11 | 0.59[0.13] | 11 | 0.70[0.11] | 10 | 0.73[0.20] | 54 | 0.64[0.14] | 1.38 | 0.064*** | Non-dominance |
| Equipment availability index, *** | 11 | 0.53[0.13] | 11 | 0.61[0.16] | 11 | 0.63[0.15] | 11 | 0.79[0.14] | 10 | 0.77[0.21] | 54 | 0.66[0.18] | 1.45 | 0.087*** | Non-dominance |
| Medical supplies available index, ** | 11 | 0.57[0.08] | 11 | 0.69[0.09] | 11 | 0.68[0.14] | 11 | 0.66[0.15] | 10 | 0.71[0.23] | 54 | 0.66[0.15] | 1.25 | 0.027 | Non-dominance |
| Contraceptive available index, *** | 11 | 0.47[0.15] | 11 | 0.49[0.19] | 11 | 0.57[0.18] | 11 | 0.77[0.09] | 10 | 0.76[0.10] | 54 | 0.61[0.19] | 1.62 | 0.117*** | Non-dominance |
| Overall– excluding staff, *** | 11 | 0.50[0.05] | 11 | 0.60[0.07] | 11 | 0.57[0.11] | 11 | 0.70[0.10] | 10 | 0.74[0.17] | 54 | 0.62[0.13] | 1.48 | 0.076*** | Non-dominance |

* Significant at 10%; ** significant at 5%; *** significant at 1% : reflects the differences between quintiles.

Note: Note: D- = 45 degree line dominates; D+ = Concentration curve dominates

Table S3b: Descriptive Statistics by Distance

| Distance | Most remote  (68 – 172)KMS | | Second most remote | | Middle | | Second least remote | | Least remote  (1 – 13) KMS | | Overall | | Equity ratio |
| --- | --- | --- | --- | --- | --- | --- | --- | --- | --- | --- | --- | --- | --- |
| Variables | n | Mean[std] | n | Mean[std] | n | Mean[std] | n | Mean[std] | n | Mean[std] | n | Mean[std] |  |
| Clinical staff per 1,000 population | 10 | 0.22[0.27] | 11 | 0.19[0.23] | 9 | 0.25[0.22] | 13 | 0.34[0.24] | 11 | 0.34[0.32] | 54 | 0.27[0.26] | 1.56 |
| Nurses per 1,000 population | 10 | 0.25[0.21] | 11 | 0.54[0.38] | 9 | 0.38[0.35] | 13 | 0.54[0.44] | 11 | 0.48[0.35] | 54 | 0.45[0.36] | 1.90 |
| Support staff per 1,000 population | 10 | 0.25[0.29] | 11 | 0.31[0.29] | 9 | 0.28[0.27] | 13 | 0.17[0.19] | 11 | 0.17[0.20] | 54 | 0.23[0.25] | 0.67 |
| Total staff per 1,000 population | 10 | 0.72[0.67] | 11 | 1.03[0.61] | 9 | 0.90[0.60] | 13 | 1.05[0.64] | 11 | 0.99[0.75] | 54 | 0.95[0.64] | 1.37 |
| Vaccination availability index, * | 10 | 0.90[0.32] | 11 | 1.00[0.00] | 9 | 0.98[0.06] | 13 | 1.00[0.00] | 11 | 0.99[0.05] | 54 | 0.98[0.14] | 1.10 |
| Antibiotics availability index , ** | 10 | 0.63[0.17] | 11 | 0.58[0.19] | 9 | 0.56[0.12] | 13 | 0.73[0.20] | 11 | 0.65[0.20] | 54 | 0.64[0.19] | 1.03 |
| Anti-malarial availability index | 10 | 0.93[0.14] | 11 | 0.97[0.10] | 9 | 0.96[0.11] | 13 | 0.92[0.15] | 11 | 0.85[0.31] | 54 | 0.93[0.18] | 0.91 |
| Oxytocics -availability index | 10 | 0.37[0.19] | 11 | 0.52[0.27] | 9 | 0.37[0.20] | 13 | 0.49[0.26] | 11 | 0.49[0.31] | 54 | 0.45[0.25] | 1.22 |
| Anti-hypertensive drugs availability index | 10 | 0.33[0.21] | 11 | 0.36[0.21] | 9 | 0.31[0.21] | 13 | 0.44[0.34] | 11 | 0.52[0.33] | 54 | 0.40[0.27] | 1.21 |
| ART availability index, * | 10 | 0.21[0.32] | 11 | 0.23[0.28] | 9 | 0.37[0.42] | 13 | 0.43[0.39] | 11 | 0.67[0.35] | 54 | 0.39[0.38] | 3.19 |
| Anti-diarrhoeal index | 10 | 0.70[0.35] | 11 | 0.64[0.32] | 9 | 0.67[0.25] | 13 | 0.77[0.26] | 11 | 0.55[0.42] | 54 | 0.67[0.32] | 0.96 |
| Other drugs | 10 | 0.54[0.28] | 11 | 0.62[0.19] | 9 | 0.76[0.24] | 13 | 0.60[0.29] | 11 | 0.82[0.19] | 54 | 0.66[0.26] | 1.22 |
| Total drugs , * | 10 | 0.58[0.13] | 11 | 0.61[0.12] | 9 | 0.62[0.10] | 13 | 0.67[0.13] | 11 | 0.69[0.19] | 54 | 0.64[0.14] | 1.10 |
| Equipment availability index, ** | 10 | 0.56[0.15] | 11 | 0.59[0.12] | 9 | 0.73[0.20] | 13 | 0.70[0.20] | 11 | 0.73[0.20] | 54 | 0.66[0.18] | 1.30 |
| Medical supplies available index | 10 | 0.63[0.11] | 11 | 0.66[0.09] | 9 | 0.59[0.11] | 13 | 0.69[0.17] | 11 | 0.72[0.20] | 54 | 0.66[0.15] | 1.14 |
| Contraceptive available index, *** | 10 | 0.56[0.14] | 11 | 0.50[0.20] | 9 | 0.57[0.23] | 13 | 0.62[0.18] | 11 | 0.78[0.12] | 54 | 0.61[0.19] | 1.39 |
| Overall– excluding staffs , ** | 10 | 0.56[0.09] | 11 | 0.58[0.10] | 9 | 0.60[0.12] | 13 | 0.65[0.14] | 11 | 0.70[0.17] | 54 | 0.62[0.13] | 1.25 |

* Significant at 10%; ** significant at 5%; *** significant at 1%: reflects the difference between quintile

Annex

Annex 1: Household Ownership of Properties

| 1 | How many sleeping rooms do you have in your household? | | | | | |_____| | | |
| --- | --- | --- | --- | --- | --- | --- | --- | --- |
| 2 | What is the MAIN source of drinking water for members of your household?  [DO NOT READ LIST. CODE ONLY ONE RESPONSE] | | PIPED WATER  Piped into dwelling *...............01*  Piped into yard/plot *.............02*  Public/neighbours *................03*  COVERED WELL / BOREHOLE  In dwelling*.....................04*  In yard/plot *....................05*  Public/neighbours well  *.......06*  OPEN WELL  In dwelling*......................07*  In yard/plot*.....................08*  Public /neighbours well  *......09*  SURFACE WATER  Spring *................................10*  River/dam/lake/pond/stream/  Canal*................................11*  OTHER  Rain water *........................12*  Tanker truck *......................13*  Water vendor *………….........14*  Bottled water *...................15*  OTHER (specify) __________88 | | | | | CODE  |__|__| |
| 3 | What kind of toilet facility does your household usually use?  [DO NOT READ LIST. CODE ONLY ONE RESPONSE] | | Flush to piped sewer *…..........01*  Flush to piped septic tank....... *02*  Flush to pit latrine *.................03*  Flush to elsewhere *.......………04*  Ventilated improved pit latrine*.05*  Pit latrine with slab *.........….06*  Pit latrine without slab/open  *.07*  Composting toilet/ecoscan *….08*  Bucket *.....................…………09*  No facility/bush/field *....…..10*  Other (specify) *___________*88 | | | | | CODE  |__|__| |
| 4 | What type of fuel does your household MAINLY use for cooking?  [DO NOT READ LIST. CODE ONLY ONE RESPONSE] | | Electricity *.........................01*  Solar *......................02*  Gas *...........................03*  Paraffin/kerosene *..............04*  Charcoal *......................05*  Firewood  *....................06*  Crop residuals/straw/grass  /animal dung *..................07*  No cooking facilities in Household *08*  Other (specify) *_______*88 | | | | | CODE  |__|__| |
| 5 | What is the MAIN source of energy for lighting in the household?  [DO NOT READ LIST. CODE ONLY ONE RESPONSE] | | Electricity *........................01*  Solar *................................02*  Gas *...................................03*  Paraffin/kerosene  *...........04*  Candle *............................05*  Firewood *..........................06*  *Torch/battery ....................07*  Other (specify) Chanzo  *.........88* | | | | | CODE  |__|__| |
| 6 | What is the main material of the floor of your house?  [DO NOT READ LIST. CODE ONLY ONE RESPONSE] | | Earth/sand/dung...............01  Wood planks ...............02  Bamboo/palm *...............*03  Parquet or polished wood *..*04  Vinyl or asphalt strips.........05  Ceramic tiles *..................*06  Cement *..........................*07  Carpet *..........................*08  Other (specify) *.................*88 | | | | | CODE  |__|__| |
| 7 | What is the main material of your roof?  [DO NOT READ LIST. CODE ONLY ONE RESPONSE] | | No roof *……………………01*  Grass/leaves/palm/bamboo  /mud *…………………………...02*  Rustic mat…………….......…….03  Metal sheets *………*……......04  Concrete/cement *……….*….05  Wood *.*………….............…….06  Asbestos…………………….…….07  Ceramic tiles *………..*………….08  Other (specify) *....*..............88 | | | | | CODE  |__|__| |
| 8 | What is the main material of your walls?  [DO NOT READ LIST. CODE ONLY ONE RESPONSE] | | | No walls ………………….…..01  Grass/poles/mud *….......*…02  Bamboo with mud *…..…*…..03  Stones with mud *………..…..*04  Wood/timber *………*……......05  Cardboard *…*……………..........06  Mud or sundried Bricks ...….07  Baked/burnt bricks …….......08  Cement blocks ....................09  Concrete or stone bricks .....10  Other (specify) *_______*____88 | | | | CODE  |__|__| |
| 9 | Does your household have electricity? | | | YES *...........................01*  NO *.........................02* | | | | CODE  |__|__| |
| 10 | Does your household own a radio in working order? | | | YES *...............01*  NO *................02* | CODE  |__|__| | | | |
| 11 | Does your household own a TV in working order? | | | YES *.............. 01*  NO *..................02* | CODE  |__|__| | | | |
| 12 | Does your household own a DVD player in working order? | | | YES *..............01*  NO *............02* | CODE  |__|__| | | | |
| 13 | Does your household own a mobile phone in working order? | | | YES *.............01*  NO *............02* | CODE  |__|__|  If no, skip to 7.21 | | | |
| 14 | How many mobile phones that are in working order are owned by your household? | | | | | | | |_____| |
| 15 | Does your household own a land line? | | | YES *.............01*  NO *..........02* | | | CODE  |__|__| | |
| 16 | Does your household own an iron that is in working order? | | | YES *...........01*  NO *..........02* | | | CODE  |__|__| | |
| 17 | Does your household own a refrigerator that is in working order? | | | YES *............01*  NO *...........02* | | | CODE  |__|__| | |
| 18 | Does your household own a watch that is in working order? | | | YES *............01*  NO *.........02* | | | CODE  |__|__| | |
| 19 | Does your household own a sewing machine that is in working order? | | | YES *............01*  NO *.........02* | | | CODE  |__|__| | |
| 20 | Does your household own a table? | | | YES *............01*  NO *.........02* | | | CODE  |__|__|  If no, skip to 7.28 | |
| 21 | How many tables do your household own? | | | | | | |_____| | |
| 22 | Does your household own a sofa? | | | YES *..............01*  NO *...........02* | | | CODE  |__|__|  If no, skip to 7.30 | |
| 23 | How many sets of sofas do your household own? | | | | | | |_____| | |
| 24 | Does your household own a cupboard | | | YES *...............01*  NO *............02* | | | CODE  |__|__|  If no, skip to 7.32 | |
| 25 | How many cupboards does your household own? | | | | | | |_____| | |
| 26 | Does your household own a motorcycle? | YES *..............01*  NO *.......02* | | | | | CODE  |__|__| | |
| 27 | Does your household own a car? | YES *..............01*  NO *............02* | | | | | CODE  |__|__| | |
| 28 | Does any member of your household have a bank account? | YES *...............01*  NO *...........02* | | | | | CODE  |__|__| | |

Annex 2: Health Facility Survey

2.1 Vaccination

| 1606 | Vaccine: *verification of the vaccine availability was done and register/stock cards were checked if there was a stock out in the past 90 days.* | | |
| --- | --- | --- | --- |
|  | Vaccine | Vaccine available?  Use:  Yes ….............1  No ……………...0 | Stock out in last 90 days  Use:  Yes ……………………..1  No …………………….0  INAP ………………..77  Don’t know  *………*…99 |
| A | Tetanus toxoid (child) | [ ] | [ ] |
| B | BCG and dilutant | [ ] | [ ] |
| C | Oral polio (OPV) | [ ] | [ ] |
| D | DPT | [ ] | [ ] |
| E | Measles and dilutant | [ ] | [ ] |
| F | Vitamin A | [ ] | [ ] |

2.2 Drugs/medicines

| 1608 | Medicine/ Drug | a) Medicine available?  Use :  Yes ….1  No …..0 | b) Stock out in last 90 days  Use:  No ……….. ..0  Yes  ………… ..1  INAP …..77  Don’t know …99 |  |
| --- | --- | --- | --- | --- |
| A | Cotrimo-xazole | [ ] | [ ] |  |
| B | Artemeter Lumefantrine (ALu) | [ ] | [ ] |  |
| C | Quinine | [ ] | [ ] |  |
| D | Oral Rehydration Salts | [ ] | [ ] |  |
| E | Zinc tablets | [ ] | [ ] |  |
| F | Normal Saline (NS) | [ ] | [ ] |  |
| G | Aldomet | [ ] | [ ] |  |
| H | Hydralazine | [ ] | [ ] |  |
| I | Rtize ant TB RHZE | [ ] | [ ] |  |
| J | Oxytocin | [ ] | [ ] |  |
| K | Ergometrine | [ ] | [ ] |  |
| L | Misoprostol | [ ] | [ ] |  |
| M | Diazepam/Valium | [ ] | [ ] |  |
| N | Magnesium Sulfate | [ ] | [ ] |  |
| O | SP (IPTp) | [ ] | [ ] |  |
| P | Flagyl | [ ] | [ ] |  |
| Q | Gentamycin | [ ] | [ ] |  |
| R | Ampicilline | [ ] | [ ] |  |
| S | Chloramphenical | [ ] | [ ] |  |
| T | X-pen | [ ] | [ ] |  |
| U | Savlon | [ ] | [ ] |  |
| V | Povidone iodine | [ ] | [ ] |  |
| W | Eye drops/ iodine | [ ] | [ ] |  |
| X | Ziduvidine | [ ] | [ ] |  |
| Y | Stavudine | [ ] | [ ] |  |
| Z | Lamivudine | [ ] | [ ] |  |
| AA | Combination of A, C & G | [ ] | [ ] |  |
| AB | Emtricitabine | [ ] | [ ] |  |
| AC | Tenofovir | [ ] | [ ] |  |
| AD | Nevirapine | [ ] | [ ] |  |
| AE | Efavirenz | [ ] | [ ] |  |

2.3 Equipments

| 1612 | Records on equipment functioning and not functioning | | |
| --- | --- | --- | --- |
|  | a) Equipment | b) No. functioning  *None ------ 00*  *Don’t know -- 99* | c) Problems functioning in past 90 days  Yes  1  No 0  INAP  77  Don’t know  99 |
| A | Sphygmomanometer (blood pressure apparatus) | [ ] | [ ] |
| B | Stethoscope | [ ] | [ ] |
| C | Time or watch | [ ] | [ ] |
| D | Infant and/ or child weighing scale | [ ] | [ ] |
| E | Measuring tape for MUAC | [ ] | [ ] |
| F | Test kit for hemacrit or hemoglobin | [ ] | [ ] |
| G | Re-agents for test kit for hemacrit or hemoglobin | [ ] | [ ] |
| H | Neonatal ambu-bag and mask | [ ] | [ ] |
| J | Autoclave equipment | [ ] | [ ] |
| M | Mucus suction apparatus | [ ] | [ ] |
| N | Delivery kits | [ ] | [ ] |
| O | Delivery table | [ ] | [ ] |
| Q | Thermometer | [ ] | [ ] |
| R | Examination lamp/torch | [ ] | [ ] |
| S | Stainless steel bowls, kidney dishes | [ ] | [ ] |
| T | Microscope | [ ] | [ ] |

2.4 Medical Supplies:

| 1613 | a. Supply | b. Available?  Use :  Yes  *…*.1  No …..0 | C. Stock out in last 90 days  Yes  1  No 0  INAP  77  Don’t know 99 |
| --- | --- | --- | --- |
| A | Sterile latex gloves | [ ] | [ ] |
| B | Disinfectant (mention) | [ ] | [ ] |
| C | Cotton wool | [ ] | [ ] |
| D | Malaria Rapid Diagnostic Tests (MRDT) Kits | [ ] | [ ] |
| E | Glass slide malaria test | [ ] | [ ] |
| F | Partograph | [ ] | [ ] |
| G | Sutures | [ ] | [ ] |
| H | Urine catheters | [ ] | [ ] |
| I | Suction catheters | [ ] | [ ] |
| J | Oxygen supply | [ ] | [ ] |
| K | Gas supply (for vaccine refrigeration) | [ ] | [ ] |

2.5 Contraceptives

|  | FP Product | 1. Available?   Use :  Yes  *…*.1  No …..0 | 1. Stock out in last 90 days   Yes  1  No 0  INAP 77  Don’t know  99 |
| --- | --- | --- | --- |
| A | Male condom | [ ] | [ ] |
| B | Female Condom | [ ] | [ ] |
| C | Combined oral Pill | [ ] | [ ] |
| D | Progestin only pill | [ ] | [ ] |
| E | Depo-Provera | [ ] | [ ] |
| F | 1-mon. / combined injectable | [ ] | [ ] |
| G | IUCD (any type) | [ ] | [ ] |
| H | Implants (any type – Norplant, Implanon, Jadelle) | [ ] | [ ] |

Annex 3: Health care reforms

| **Reform** | **Description** |
| --- | --- |
| Change of CHF/TIKA management, from MOHSW to NHIF. | In early 2009, the NHIF took over the management of the CHF from the Ministry of Health and Social Welfare (MoHSW) initially for a 3 year period, a first step towards the merger of these schemes. The NHIF entered into a memorandum of Understanding with the MoHSW and the Prime Minister’s Office for Regional Administration and Local Government (PMORALG). The objectives were to harmonise the NHIF and CHF management operations, by incorporating CHF management structures within NHIF, to improve efficiency and supervision, to increase awareness of the CHF and to increase coverage in line with universal coverage objectives. |
| The introduction of TIKA in urban districts. | TIKA[[1]](#footnote-2) is a Community Health Fund (CHF) for the urban informal sector. It is a voluntary health insurance scheme aiming to make health services more affordable and accessible to those living in the informal economy. Whilst the CHF has been in existence for many years in rural areas, there has so far been very little experience with voluntary health insurance in uban areas. The NHIF have re-designed TIKA, and initial plans are underway to introduce the scheme in Ilala, Kinondoni, Kibaha and Babati councils. It is expected that TIKA will later be scaled up to urban areas across the country. |
| Facility construction and subsequent staffing and equipment under the MMAM. | Facility construction is part of the MMAM[[2]](#footnote-3) program. The MMAM’s overall objective is to facilitate the provision of primary health care for all by improving access to primary health services. |
| Introduction of facility bank accounts. | Historically, health centers and dispensaries in Tanzania have not had bank accounts, rather cost sharing funds have been held at the district level. However, starting in 2007-2008 with the introduction of the MMAM and subsequently a Pay for Performance pilot, there has been growing pressure for primary level facilities to open their own bank accounts. The rationale for this is to facilitate access to funds for facilities for minor restoration and purchase of equipment, drugs and medical supplies, and to increase awareness of cost sharing fund availability |
| Service agreements as part of the public private partnership policy. | Service Agreements (SA) is one of the components of the public private partnership policy. The SA is a mechanism through which district councils enter into contract with the private sector (generally faith-based providers) to provide health services to the public and especially vulnerable groups. In 2008 the PMORALG released a circular to all the mainland regions and Local Government Authorities (LGAs) to start entering into agreement with private service providers for the purposes of supporting the MMAM. The aim of the SA is to increase the availability of accessible, quality health services. |

1. TIKA is an abbreviation of Swahili word “Tiba Kwa Kadi” that refers to treatment by health Card [↑](#footnote-ref-2)
2. Primary Health Service development Programme (PHSDP) 2007-2017 or often known as Mpango wa Maendeleo wa Afye ya Msingi (MMAM) in swahili [↑](#footnote-ref-3)
